# A Population‐Based Correlation Analysis Between Hemoglobin A1c and Hemoglobin Levels

**DOI:** 10.1111/1753-0407.70057

**Published:** 2025-02-20

**Authors:** Tingyu Zhang, Tianyi Shi, Min Cao, Yunxi Ji, Yanbin Xue, Huayan Yao, Qiaomei Yin, Bin Cui, Zhen Xie, Ping He

**Affiliations:** ^1^ Department of Endocrine and Metabolic Diseases, Shanghai Institute of Endocrine and Metabolic Diseases, Ruijin Hospital Shanghai Jiao Tong University School of Medicine Shanghai China; ^2^ Kunming First People's Hospital Yunnan China; ^3^ Faculty of Medical and Health Sciences University of Auckland Auckland New Zealand; ^4^ Computer Net Center, Ruijin Hospital Shanghai Jiao Tong University School of Medicine Shanghai China; ^5^ Department of Dermatology, Sichuan Provincial People's Hospital University of Electronic Science and Technology of China Chengdu China; ^6^ Chinese Academy of Sciences Sichuan Translational Medicine Research Hospital Chengdu China; ^7^ Link Healthcare Engineering and Information Department Shanghai Hospital Development Center Shanghai China

**Keywords:** estrogen, hemoglobin, hemoglobin A1c

## Abstract

**Background:**

Glycated hemoglobin (HbA1c) is widely used to assess long‐term glycemic control in individuals with diabetes. However, various conditions that affect hemoglobin levels or the lifespan of red blood cells can compromise the accuracy of HbA1c measurements. Despite extensive research, the relationship between HbA1c and hemoglobin remains unclear. This study aims to clarify this relationship by examining its correlation across diverse age and gender cohorts.

**Methods:**

Data from 217,991 participants aged 20 to 69 years were collected from health examination centers in Southwest China. Standardized methodologies were used to measure HbA1c and hemoglobin levels. Generalized additive models (GAM) were utilized to analyze non‐linear relationships and adjust for potential confounding variables. Gender‐specific reference intervals (RIs) for hemoglobin were also established.

**Results:**

A gender‐specific association was observed between HbA1c and hemoglobin levels. In men, HbA1c levels decreased with increasing hemoglobin. Among women, a negative correlation was observed in premenopausal women (aged ≤ 45 years), whereas postmenopausal women (aged > 45 years) showed a positive correlation, with HbA1c levels increasing as hemoglobin levels rose. Additionally, HbA1c levels increased with age in both genders, with a more pronounced rise in women after the age of 45.

**Conclusion:**

This study highlights significant gender‐ and age‐related differences in the relationship between HbA1c and hemoglobin. The findings suggest that estrogen‐related metabolic changes may influence HbA1c levels, with potential implications for diabetes management and hormone therapy in postmenopausal women.

## Graphical abstract

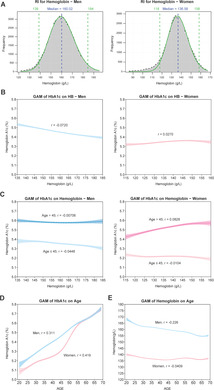

1

**Summary**

- This study investigated the association between HbA1c and hemoglobin levels in a large population cohort, revealing gender‐specific patterns across different age groups.
- A weak negative correlation was observed between HbA1c and hemoglobin in men and in women under 45 years, whereas a positive correlation was noted in women over 45 years.
- The findings suggest that estrogen may influence HbA1c levels, especially in postmenopausal women, potentially informing strategies for diabetes management and hormone‐related therapies.

To the Editor,

1

The glycated hemoglobin (HbA1c) is widely used for monitoring diabetes and guiding management decisions as it reflects long‐term blood glucose levels rather than instantaneous readings. HbA1c is formed by the attachment of glucose to N‐terminal valine residue of the hemoglobin β‐chain in a non‐enzymatic manner. A level of HbA1c ≥ 6.5% is recommended as the diagnostic threshold for diabetes. However, certain medical conditions that affect red blood cell lifespan or hemoglobin can impact the accuracy of the HbA1c measurements [1]. While there have been several studies on the relationship between HbA1c and hemoglobin, the conclusions remain unclear and inconsistent. Some studies have reported a positive correlation between HbA1c and hemoglobin [2, 3], whereas others have observed a negative correlation [4, 5]. This study undertakes a population‐based analysis to investigate the correlation between HbA1c and hemoglobin across different age and genders. By addressing these uncertainties, it aims to enhance the interpretative accuracy of HbA1c, thereby improving its applicability in clinical decision‐making across diverse patient populations.

## 2 Methods

The research clinical data, including 217 991 individuals, were collected from health examination centers of Sichuan Provincial People's Hospital and Kunming First People's Hospital, located in Southwest China. The biochemical test results were extracted from electronic health records. Both hospitals used the same detection methods: HbA1c concentrations were measured using the Advia 2400 analyzer (Siemens Healthineers, Germany) through the immunoturbidimetric method, while hemoglobin levels were determined using the colorimetric method with the DH‐615 analyzer (DIRUI Industrial Co., China).

Data cleaning, statistical analyses, and figure plot were conducted using R language (version 4.3.1). Reference intervals (RIs) for hemoglobin levels were established by gender using the refineR algorithm with refineR package (version 1.6). Unlike traditional indirect algorithms that use a forward modeling approach, the refineR algorithm stands out by employing an inverse modeling strategy to enhance the precision of reference interval estimation [6]. Additionally, we leveraged generalized additive models (GAM) to capture the flexibility required for modeling non‐linear relationships and addressing non‐normally distributed data [7].

## 3 Results

**FIGURE 1:**
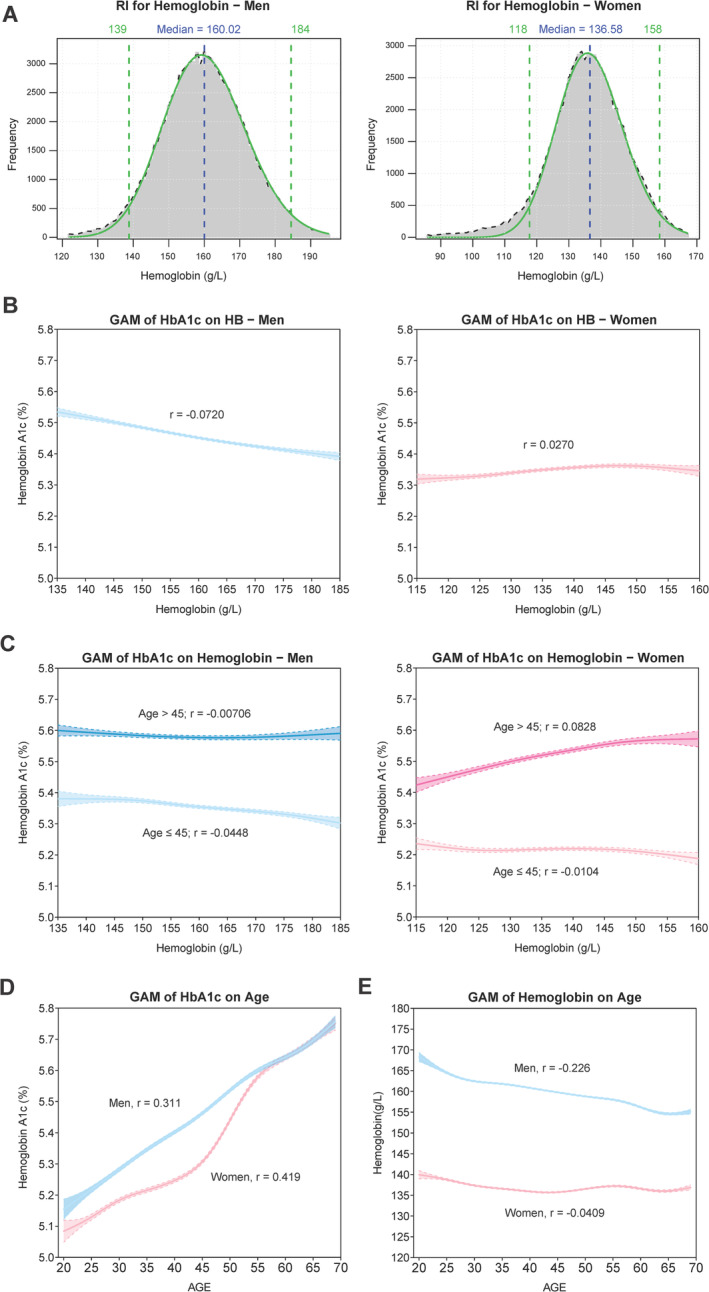
The correlation between hemoglobin A1c and hemoglobin. (A) Optimal parametric models for hemoglobin in men and women calculated using the refineR algorithm. (B, C) Generalized additive models (GAMs) depicting the relationship between hemoglobin A1c and hemoglobin, fitted with smoothing splines for men (blue) and women (pink). The shaded regions represent 95% confidence intervals. (D, E) GAMs illustrating the relationships between both hemoglobin A1c and hemoglobin with age, modeled using smoothing splines for men (blue) and women (pink). r, Pearson's correlation coefficient.

Excluding potential confounding factors that may skew statistical analysis, a total of 172 985 individuals aged 20–69 were included in our study (Figure S1). Table 1 shows general characteristics of the study population. The results showed the RIs of hemoglobin level was 139–184 g/L for man and 118–158 g/L for woman, which closely aligned with WHO criteria [8] (Figure 1A). To model the non‐linear relationships, smoothing splines were integrated into the GAM framework to generate graphical representations of median values, offering more precise insights based on real‐world data. Gender was found to have significant effects on the association between HbA1c and hemoglobin. In men, HbA1c values decreased with higher hemoglobin levels, while in women, there was a marginal increase (Figure 1B). For further analysis, HbA1c levels were observed to increase with age in both genders, with a more noticeable increase in women after the age of 45 (Figure 1D). Conversely, hemoglobin levels decreased with age in both men and women, stabilizing in women after the age of 45 (Figure 1E). An additional analysis was conducted to explore the correlation between HbA1c and hemoglobin specifically among women in different age groups, using 45 years old as a cut‐off point. For younger women (≤ 45 years old), HbA1c values marginally decreased with higher hemoglobin levels, but significantly increased in women over 45 years old (Figure 1C).

**TABLE 1:** General characteristics of the study population.

	Total	Female	Male	*p*	Cohen's *d* value
Number	172985	79762	93223		
Age, years	43.74 ± 11.25	43.31 ± 11.31	44.10 ± 11.18	< 0.001	−0.0710
BMI, kg/m^2^	23.57 ± 3.28	22.43 ± 3.08	24.55 ± 3.12	< 0.001	−0.681
SBP, mm Hg	116.43 ± 14.84	112.96 ± 15.11	119.40 ± 13.94	< 0.001	−0.444
DBP, mm Hg	72.14 ± 10.25	69.15 ± 9.74	74.69 ± 9.98	< 0.001	−0.560
Hemoglobin A1c, %	5.41 ± 0.43	5.35 ± 0.41	5.46 ± 0.44	< 0.001	−0.257
FPG, mmol/L	4.98 ± 0.57	4.94 ± 0.53	5.02 ± 0.60	< 0.001	−0.140
White blood cell count, 10^9^/L	6.04 ± 1.61	5.71 ± 1.53	6.33 ± 1.62	< 0.001	−0.397
Red blood cell count, 10^12^/L	4.92 ± 0.57	4.56 ± 0.43	5.23 ± 0.49	< 0.001	−1.470
Hemoglobin, g/L	148.58 ± 17.64	135.42 ± 12.87	159.85 ± 12.67	< 0.001	−1.914
eGFR, mL/min/1.73 m^2^	115.52 ± 25.25	127.02 ± 26.50	105.68 ± 19.28	< 0.001	0.932
TBIL, μmol/L	13.29 ± 4.74	12.26 ± 4.55	14.17 ± 4.71	< 0.001	−0.411
Total cholesterol, mmol/L	4.92 ± 0.92	4.86 ± 0.94	4.97 ± 0.91	< 0.001	−0.120
Triglycerides, mmol/L	1.59 ± 0.91	1.28 ± 0.71	1.85 ± 0.98	< 0.001	−0.658
HDL‐C, mmol/L	1.37 ± 0.34	1.52 ± 0.33	1.24 ± 0.29	< 0.001	0.881
LDL‐C, mmol/L	2.98 ± 0.83	2.84 ± 0.82	3.09 ± 0.82	< 0.001	−0.300

*Note:* Values are mean ± SD. Cohen's *d* is a statistical measure used to indicate the effect size of a result. Cohen's *d* = (M1 − M2)/SD. M1 and M2 are the means of two groups, while SD is the standard deviation of the combined group. The absolute value of Cohen's *d* can be used to measure the magnitude of the effect. The absolute value of Cohen's *d* < 0.2 is considered as a negligible effect; 0.2–0.5, a small effect; 0.5–0.8, a medium effect; ≥ 0.8, a large effect.

Abbreviations: BMI, body mass index; DBP, diastolic blood pressure; eGFR, estimated glomerular filtration rate; FPG, fasting plasma glucose; HDL‐C, high‐density lipoprotein cholesterol; LDL‐C, low‐density lipoprotein cholesterol; SBP, systolic blood pressure; TBIL, total bilirubin.

### 3.1 Comment

The inconsistencies in previous research findings could stem from variations in the male‐to‐female ratio or the age distribution of the population. In our study, we analyzed a large dataset of health examination results to explore the relationship between HbA1c and hemoglobin across different genders and age groups. The results revealed a weak negative correlation between HbA1c and hemoglobin in both men and women aged 45 and below, indicating that as hemoglobin levels increased, HbA1c levels decreased. Notably, a positive correlation was observed between HbA1c and hemoglobin in women over 45 years old. This may be attributed to the gradual decline in estrogen levels during menopause, which can lead to metabolic and endocrine changes. Estrogen appears to play a critical role in the metabolic regulation of HbA1c. Supporting this hypothesis, recent studies indicated that estrogen therapy in postmenopausal women with type 1 or type 2 diabetes can reduce HbA1c and fasting glucose levels, aligning with our findings [9].

In summary, this study revealed gender‐specific effects in the relationship between HbA1c and hemoglobin. HbA1c levels decreased with higher hemoglobin levels in men and premenopausal women, whereas in women over 45 years old, a slight increase in HbA1c was observed. These results suggest that estrogen's metabolic role may influence HbA1c levels in postmenopausal women. Our finding contributes to a deeper understanding of the complex interactions between HbA1c, hemoglobin, gender, and age, offering valuable implications for managing diabetes and hormone‐related therapies.

## Author Contributions

Q.Y. and Z.X.: contributed to data acquisition. Y.J., Y.X., and H.Y.: data cleaning. T.Z. and M.C.: analyzed and interpreted the data. T.Z. and T.S.: drafted the manuscript. T.S., M.C., and B.C.: revised the manuscript. B.C. and P.H.: conceived and designed the study. All authors agreed to be held accountable for all aspects of this work and approved the final version of the manuscript.

## Conflicts of Interest

The authors declare no conflicts of interest.

## Supporting information


**Figure S1.** Flowchart of participant inclusion in the study cohorts from Chengdu and Kunming.
