# Supplementary material for: A Population‐Based Correlation Analysis Between Hemoglobin A1c and Hemoglobin Levels
Source: J Diabetes. 2025 Feb 20;17(2):e70057. doi: 10.1111/1753-0407.70057 (PMC11842278; doi:10.1111/1753-0407.70057)
Supplement: Supplementary file 1 — Figure S1. Flowchart of participant inclusion in the study cohorts from Chengdu and Kunming. [file JDB-17-e70057-s001.pdf]

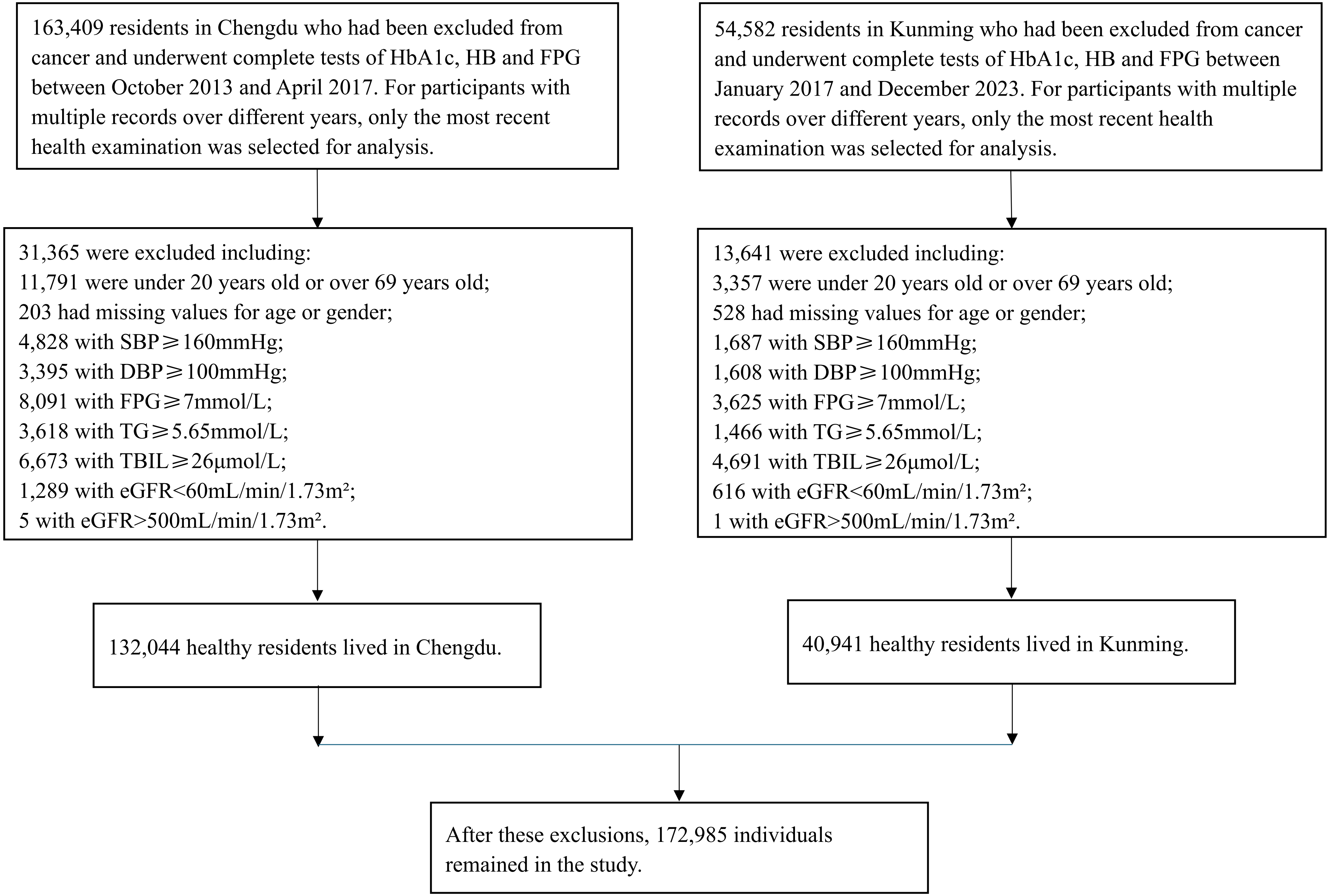
163,409 residents in Chengdu who had been excluded from cancer and underwent complete tests of HbA1c, HB and FPG between October 2013 and April 2017. For participants with multiple records over different years, only the most recent health examination was selected for analysis.
31,365 were excluded including:
11,791 were under 20 years old or over 69 years old;
203 had missing values for age or gender;
4,828 with SBP≥160mmHg;
3,395 with DBP≥100mmHg;
8,091 with FPG≥7mmol/L;
3,618 with TG≥5.65mmol/L;
6,673 with TBIL≥26μmol/L;
1,289 with eGFR<60mL/min/1.73m²;
5 with eGFR>500mL/min/1.73m².
132,044 healthy residents lived in Chengdu.
54,582 residents in Kunming who had been excluded from cancer and underwent complete tests of HbA1c, HB and FPG between January 2017 and December 2023. For participants with multiple records over different years, only the most recent health examination was selected for analysis.
13,641 were excluded including:
3,357 were under 20 years old or over 69 years old;
528 had missing values for age or gender;
1,687 with SBP≥160mmHg;
1,608 with DBP≥100mmHg;
3,625 with FPG≥7mmol/L;
1,466 with TG≥5.65mmol/L;
4,691 with TBIL≥26μmol/L;
616 with eGFR<60mL/min/1.73m²;
1 with eGFR>500mL/min/1.73m².
40,941 healthy residents lived in Kunming.
After these exclusions, 172,985 individuals remained in the study.
